# *In Vitro* and* In Vivo* Evaluation of Niosomal Formulation for Controlled Delivery of Clarithromycin

**DOI:** 10.1155/2016/6492953

**Published:** 2016-05-16

**Authors:** Gyati Shilakari Asthana, Parveen Kumar Sharma, Abhay Asthana

**Affiliations:** Department of Pharmaceutics, MM College of Pharmacy, Maharishi Markandeshwar University, Mullana, Ambala, Haryana 133207, India

## Abstract

The present study was focused on formulating and evaluating clarithromycin (CLR) containing niosomal formulation for* in vitro* and* in vivo* pharmacokinetic behavior. Niosomal formulations (empty and drug loaded) were prepared by using different ratio of surfactant (various Span grades 20, 40, 60, and 80) and cholesterol by thin film hydration method and were evaluated for* in vitro* characteristics, stability studies, and* in vivo* study. Dicetyl phosphate (DCP) was added to the niosomal formulation. Various pharmacokinetic parameters were determined from plasma of male SD rats. Span 60 containing niosomal formulation NC_2_ (cholesterol to surfactant ratio 1 : 1) displayed highest entrapment efficiency with desired particle size of 4.67 *μ*m. TEM analyses showed that niosomal formulation was spherical in shape. Niosomes containing Span 60 displayed higher percentage of drug release after 24 h as compared to other formulations. NC_2_ formulation was found to be stable at the end of the study on storage condition. Various pharmacokinetic parameters, namely, AUC, AUMC, and MRT of niosomal formulation, were found to be 1.5-fold, 4-fold, and 3-fold plain drug, respectively. The present study suggested that niosomal formulations provide sustained and prolonged delivery of drug with enhance bioavailability.

## 1. Introduction

In recent years, transporting the drug molecules to the desired site in the biological systems has become a very specific and sophisticated area of pharmaceutical research. The role of the novel drug delivery system is not only limited to a drug package convenience and ease of administration but along with this it is also needed to provide better therapeutic efficacy and safety by delivering the drug molecules to the target site in the most convenient manner. These novel carriers provide sustained drug release for prolonged duration in targeted tissue thus resulting in enhanced therapeutic efficacy and minimized side effects (as discussed by Lamprecht [1]).

Oral route represents the predominant and most preferable route for administration of therapeutic agents due to its easy formulation and economic administration. However, oral administration of drugs often leads to degradation due to the highly acidic gastric environment, enzymes of the mucosa or liver, before they enter the systemic circulation. Beside many highly polar drugs, macromolecular drugs may not be absorbed because of their insufficient poor solubility, lipophilicity, and large molecular weight (as discussed by Porter and Charman [2]; Humberstone and Charman [3]; Shively and Thompson [4]; and Bert and De Boer [5]).

Clarithromycin is a broad spectrum, second-generation macrolide antibiotic, belonging to class II drug. CLR is used in the treatment of various infections such as respiratory tract infections and skin and soft tissue infections. CLR may be given to eradicate* H. pylori *in treatment regimens for peptic ulcer diseases (as discussed by Kumar et al. [6]). It is rapidly absorbed from the gastrointestinal tract and undergoes first-pass metabolism. It causes some undesirable side effects to the gastrointestinal tract, such as diarrhea, vomiting, nausea, headache, and abdominal pain. CLR possesses poor aqueous solubility and thus suffers from poor oral bioavailability which is about 55%. The terminal half-life of CLR is reported to be 3-4 hours and hence frequent dosing is required. Thus, to obtain the control release profile of CLR, it is desirable to encapsulate the drug in the vesicular system to prolong the existence of the drug in systemic circulation and perhaps increase the bioavailability. A wide variety of carriers can be found in nature to provide control release of drug. Various novel drug delivery technologies of CLR through different route such as topical delivery of CLR emulgel (as discussed by Joshi et al. [7]), oral delivery of CLR nanoparticles (as discussed by Thagele et al. [8]), CLR microcapsule (as discussed by Hu et al. [9]), oral delivery of CLR microsphere (as discussed by Sanjivani et al. [10]) have been used.

Various novel drug delivery systems such as liposome (as discussed by Sinico et al. [11]), niosomes (as discussed by Paolino et al. [12]), nanoparticles (as discussed by Dingler et al. [13]), and microspheres (as discussed by Patel et al. [14]) have been reported to deliver the drug to the target tissues. Drug delivery system using novel vesicular carrier, such as liposome or niosome, has distinct advantages over microspheres, nanoparticles, and other carriers in terms of better entrapment of drugs (payload characteristics), better target site specificity, and handling premature drug release (burst effect). In 1985, niosomes were studied as an alternative to liposome because they offer some benefits over liposome such as being more stable, nontoxic, and economic due to low cost of nonionic surfactant as compared to phospholipids which are prone to oxidation. Incorporation of surfactants within niosomes may also enhance the efficacy of the drug, possibly by facilitating its uptake by the target cells. A surfactant used for preparation of niosomes must have a hydrophilic head and hydrophobic tail. The hydrophobic tail may consist of one or two alkyl or perfluoroalkyl groups or in some cases a single steroidal group. The ester type surfactants are chemically less stable than ether type surfactants and the former is less toxic than the latter due to ester-linked surfactant degraded by esterase to triglycerides and fatty acid* in vivo*. The surfactants with alkyl chain length from C_12_–C_18_ are suitable for preparation of niosomes (as discussed by Gopalakrishnan and Chenthilnathan [15]; Sharma et al. [16]; Shilakari et al. [17]). The presence of the steroidal molecule (cholesterol) improves the rigidity of the bilayer and is important component of the cell membrane and its presence in membrane affects bilayer fluidity and permeability. This system protects the biomolecules from the premature degradation and inactivation due to unwanted immunological and pharmacological effects. Niosomes can be prepared by hydration of synthetic nonionic surfactants either with or without cholesterol (as discussed by Barry [18]; Handjani-Vila et al. [19]). Vesicle formation is not spontaneous since it needs input of some kind of energy, for instance, via extrusion, heating, or shaking of the surfactant aqueous dispersions (as discussed by Lasic [20]). Niosomes have been studied for delivery of drug by using various routes of administration including intramuscular (as discussed by Arunothayanun et al. [21]), intravenous (as discussed by Uchegbu et al. [22]), peroral, and transdermal (as discussed by Yoshioka et al. [23]). Wide varieties of drugs such as colchicines (anti-gout) (as discussed by Hao et al. [24]), estradiol (hormone therapy) (as discussed by Fang et al. [25]), tretinoin (retinoid) (as discussed by Manconi et al. [26]), dithranol (antipsoriatic) (as discussed by Agarwal et al. [27]), enoxacin (fluoquinole antibiotic) (as discussed by Fang et al. [28]), glucocorticoid (as discussed by Marianecci et al. [29]), Beclometasone Dipropionate (BDP) (as discussed by Elhissi et al. [30]), Roxithromycin (as discussed by Saxena et al. [31]), Nifedipine (as discussed by Yasam et al. [32]), Bovine Serum Albumin (as discussed by Moghassemi et al. [33]), and Doxorubicin (as discussed by Pawar and Vavia [34]) have been delivered through niosomes. The present study involved the drug delivery potential of niosomal formulation for CLR.

In this work, niosomal formulation of clarithromycin was prepared by using different grades of Span as nonionic surfactants and was administered in male SD rats* via* pulmonary route. Various pharmacokinetic parameters, namely, *t*_max_, AUC, AUMC, and MRT of niosomal formulation, were calculated.

## 2. Materials and Methods

### 2.1. Materials

Clarithromycin was received as gift sample from Ind-swift Laboratory Industrial Area. Chloroform, acetone, potassium dihydrogen orthophosphate, sodium chloride, sodium hydroxide, methanol, disodium hydrogen phosphate, and dialysis membrane were obtained from HiMedia. Cholesterol and dicetyl phosphate (DCP) were taken from Sigma-Aldrich.

### 2.2. Determination of Solubility of CLR

Solubility of drug was determined in various solvent such as PBS 7.4, acetone, methanol, ethanol, dichloromethane, and chloroform. For this, an excess amount of drug was transferred to 50 mL volumetric flasks containing 25 mL of solvent. The volumetric flasks were securely capped and placed in the mechanical shaker water bath at 25°C for 24 hr and then sonicated for 10 min, and thus sufficient time was provided for contact to produce saturated solutions. Solutions were filtered by passing through a 0.45 *μ*m membrane filter and analyzed on UV spectrophotometer at 265 nm.

### 2.3. Preparation of Niosomal Formulation

In the present study, niosomal formulations were prepared by thin film hydration technique as reported earlier with slight modifications (as discussed by Balakrishnana et al. [35]) by using different grades of Span (Span 20, Span 40, Span 60, and Span 80) at various cholesterol : surfactant ratios, that is, 0.5 : 1, 1 : 1, 1.5 : 1, 1 : 0.5, and 1 : 1.5 (Table 1). DCP was added to the formulation that acts as a negative charge inducer which provides more efficient drug delivery and keeps the niosomal formulation stable for long period of time.

Accurately weighted quantities of surfactants and cholesterol were taken to give the desired ratio and were dissolved in 10 mL chloroform in a round bottom flask and DCP was added to the above mixture. Then, accurately weighed amount of drug was added to the solvent. The solvent was evaporated in a rotary flash evaporator under a vacuum of 20 inches of Hg at a temperature of 60°C at 120 rpm until a smooth, dry lipid film was obtained followed by introducing under high vacuum through vacuum pump for at least three hours for removal of residual content of chloroform. Further flask was kept in vacuum desiccators overnight for complete removal of chloroform (as discussed by Mukherjee et al. [36]). Then film was hydrated with 10 mL of PBS pH 7.4 for 3 hr at 60 ± 2°C with shaking on a water bath. The niosomal suspension was kept at 2–8°C for 24 hr. Developed niosomal formulation was evaluated with respect to particle size, shape, entrapment efficiency,* in vitro* drug release profile, zeta potential, polydispersity index, and stability studies.

Volume of aqueous phase was taken 10 mL. Time and temperature for hydration were 3 hr and 60 ± 2°C, respectively. These preparations were optimized on the basis of size distribution and entrapment efficiency.

## 3. Evaluation of Niosomal Formulation

Niosomes formulations were characterized with respect to shape, particle size distribution, entrapment efficiency,* in vitro* release studies, zeta potential, polydispersity index, and stability profile.

### 3.1. Particle Shape and Morphology

**Figure 1:**
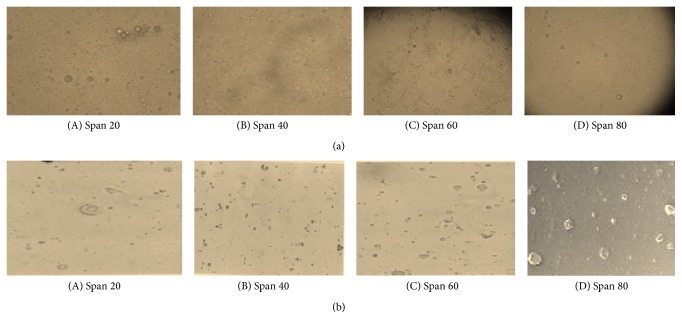
Optical microscopy of niosomes. (a) Empty and (b) drug loaded at 45x magnification.

**Figure 2:**
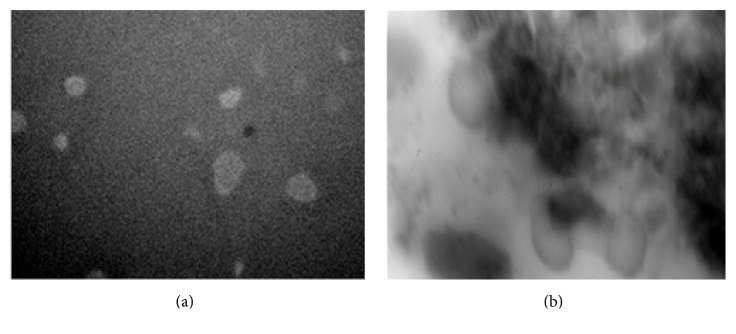
TEM image of NC_2_ formulation prepared at 1 : 1 ratio of cholesterol and Span with different magnification. (a) 40,000x and (b) 45,000x.

Shape and morphology of empty niosomal formulations and drug loaded niosomal formulations were determined by optical microscopy and Transmission Electron Microscopy (TEM) and results were shown in Figures 1 and 2.

### 3.2. Particle Size

**Figure 3:**
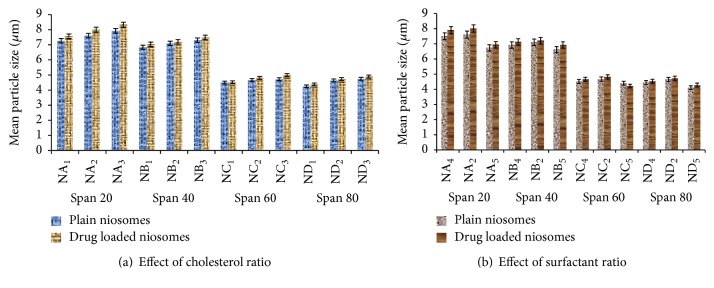
Effect of cholesterol and surfactant ratio on particle size of niosomes containing different series of Span.

A drop of niosomes suspension was placed on a glass slide. A cover slip was placed over the niosomes suspension and evaluated the average vesicle size and shape by an ordinary optical microscope using a precalibrated ocular eye piece micrometer (as discussed by Firthouse et al. [37]). Mean particle sizes of all empty niosomes formulation and drug loaded niosomal formulations were determined by using optical microscopy and results were shown in Figure 3.

### 3.3. Entrapment Efficiency

Entrapment efficiencies of niosomal formulations were determined by centrifugation method. For this, 10 mL niosomal suspension was poured into a centrifugation tube and centrifuged by using cooling centrifuged (REMI cooling centrifuge) at 10000 rpm at 4°C for 10 min.

The clear fraction was further used for the determination of free drug by using UV/visible spectrophotometer at 265 nm (as discussed by Sathali and Sangeetha [38]). The entrapment efficiency was calculated using the following formula:

(1) $$\begin{array}{c} \text{Entrapment efficiency}\left(\;\%\;\right)=\frac{\left(C_{t}-C_{f}\right)}{C_{t}}\times 100, \end{array}$$

where *C*_*t*_ is the concentration of total drug and *C*_*f*_ is the concentration of unentrapped drug.

### 3.4. *In Vitro* Release Studies

*In vitro *release pattern of niosomes suspension was carried out by dialysis bag method (as discussed by Sathali and Rajalakshmi [39]). A dialysis sac was washed and soaked in distilled water. The vesicle suspension was pipette into a bag made up of tubing and sealed followed by placing the dialysis bag into a beaker containing 200 mL of PBS pH 7.4. The vessel was placed over magnetic stirrer (50 rpm) and the temperature was maintained at 37°C ± 0.5°C. Samples were withdrawn at predetermined time intervals and immediately replaced with the fresh medium to maintain the sink condition throughout experiment. Samples were diluted and analyzed for drug content by using UV/visible spectrophotometer at 265 nm (as discussed by Sathali and Rajalakshmi [39]).

### 3.5. Method

See Table 1.

### 3.6. Zeta Potential and Polydispersity Index

Zeta potential and polydispersity index of the NC_2_ niosomal formulation were measured by using Malvern Zetasizer Nano ZS and results were shown in Table 3. The polydispersity index of niosomes was performed as a measurement of size distribution of the delivery system.

### 3.7. Transmission Electron Microscopy (TEM)

Transmission electron microscopy (TEM) was used to determine the morphology of the niosomal vesicles. Few drops of optimized niosomal formulation (NC_2_) were deposited on a carbon-coated copper grid and examined under transmission electron microscope.

### 3.8. Cell Cytotoxicity Study

Cell cytotoxicity study of drug loaded niosomal formulation and plain drug was determined on human lung carcinoma cell line (A549) by using MTT assay (as discussed by Asthana et al. [40] and Wadhwa et al. [41]). The cell growth inhibition activity of samples was evaluated by 3-(4,5-dimethylthiazol-2-yl)-2,5-diphenyltetrazolium bromide (MTT) colorimetric assay. Briefly, cell lines were maintained in Dulbecco's Modified Eagle's Medium (DMEM) under suitable conditions. The cells were cultured in Dulbecco's Modified Eagle's Medium (DMEM) containing 10% heat-inactivated FCS and supplemented with 100 U/mL of penicillin and 100 *μ*g/mL streptomycin at 37°C in a humidified atmosphere with 5% CO_2_ in air. The cells were seeded onto 24-well microtiter plates (0.1 mL/well, containing 5 × 10^3^ cells/well) and incubated for 24 hr at 37°C. Twenty-four hours later, the old medium was carefully aspirated and the cells were incubated in a logarithmic growth phase with various concentrations of plain drug solution, equivalent CLR loaded niosomal formulation (NC_2_), and empty niosomal formulation. After 24 h of incubation, the old medium was aspirated and replaced with fresh medium (DMEM). After incubation, 30 *μ*L of 5 mg/mL MTT dye solution (in PBS) was added to each well and the plate was incubated for another 3 hr at 37°C allowing viable cells to reduce the MTT into purple colored formazan crystal. Following incubation, the culture medium was removed from wells by slow aspiration and 50 *μ*L of dimethyl sulfoxide (DMSO) was added to each well to dissolve the formazan crystals and the plate was incubated for 30 min at room temperature. Optical density was measured using a microplate reader at 550 nm. The cell viability was expressed as percentage compared to a control that had not been treated with polymers, using the following equation:

(2) $$\begin{array}{c} \text{Cell Viability}\left(\;\%\;\right)=\frac{\left(OD590\;\;sample\right)}{\left(OD590\;\;control\right)}\times 100, \end{array}$$

where the OD590 sample represents the measurement from the wells treated with polymer and OD590 control represents the wells treated with PBS buffer only.

### 3.9. Stability Studies

The purpose of stability testing is to provide evidence on how the quantity of a drug substance or drug product varies with time under the influence of a variety of environmental factors such as temperature, humidity, and light and to establish a retest period for the drug substance or a shelf life for the drug product and recommended storage conditions.

On the basis of the results of* in vitro* characterization of the developed niosomal formulation, NC_2_ (1 : 1 cholesterol and surfactant ratio) formulation was selected for further stability study and* in vivo *study. Stability study of NC_2_ formulation was carried out by assessing the ability of vesicles to retain the drug (Drug Retention Behavior). NC_2_ niosomal formulation was kept at two different temperature conditions, that is, refrigeration temperature and room temperature (RT). Throughout the study, niosomal formulations were stored in aluminum foil-sealed glass vials. Samples were withdrawn at the 7th, 14th, 21st, 28th, and 30th day and were examined for physical changes such as color, particle size, and residual drug content spectrophotometrically (as discussed by Jadon et al. [42]).

### 3.10. *In Vivo* Studies

Male SD rats weighing about 200–250 g were taken for* in vivo* study. They were allowed free access to water but forbidden to eat 12 hr before the experiment. The approval of the institutional animal ethical committee (MMCP/IAEC/12/7) was obtained before starting the study. Animals were divided into 3 groups and each group contains 5 animals. The first group was treated as control and pure drug and NC_2_ formulation in aerosolized form (0.2 mg/kg) were administered to the 2nd and 3rd group of animals, respectively. Blood samples were withdrawn from each animal into heparinised tubes at different interval of time as 0, 1, 2, 4, 6, 8, 12, and 24 hr. Plasma was separated and drug concentration was determined by UV spectrophotometer at 265 nm. After that different pharmacokinetic parameter such as *t*_max_, area under curve (AUC), area under mean curve (AUMC), and mean residence time (MRT) were to be calculated from the data as shown in Table 5.

## 4. Result and Discussion

### 4.1. Solubility Determination

Solubility of drug was determined by dissolving the drug in different solvents such as water, PBS pH 7.4, methanol, acetone, dichloromethane, chloroform, and ethanol. The result as shown in Table 2 revealed that solubility of CLR in dichloromethane was highest whereas the lowest solubility was recorded in PBS 7.4.

## 5. Evaluation of Niosomal Formulation

Developed niosomal formulations were characterized with respect to particle size, shape, entrapment efficiency, and* in vitro *drug release profile.

### 5.1. Shape and Morphology

Shape and morphology of niosomal formulations were determined by optical microscopy. It was clearly observed from Figure 1 that niosomes are spherical in shape.

### 5.2. Transmission Electron Microscopy (TEM)

Morphological characteristics of niosomal formulations were further confirmed by TEM analysis. TEM photomicrograph of (NC_2_) niosomal formulation at 40,000x (Figure 2(a)) and 45,000x (Figure 2(b)) magnification revealed the spherical shape and morphology of the niosomes. Further, it was observed from the TEM images that niosomes are with hollow vesicular structure.

### 5.3. Particle Size

Particle size of the various developed niosomal formulation containing different grade of Span was determined by optical microscopy. It was clearly observed from the result as shown in Figure 3 that mean vesicle size empty niosomes containing Span 20 were found to be higher as compared to other niosomes containing different grade of Span such as Span 40, Span 60, and Span 80. The particle sizes of niosomal formulation were reported in the range of 4 *μ*m to 8 *μ*m in case of empty niosomes. The particle sizes of niosomes were decreased consistently from Span 20 to Span 80 and are found in the following order:

(3) $$\begin{array}{c} \text{Span }20>\text{Span }40>\text{Span }60>\text{Span }80. \end{array}$$

This might be due to the increase in the hydrophobicity of the surfactant from Span 20 to Span 80. The decrease in surface free energy with increasing the hydrophobicity of surfactants may be the major attribute of reduction in the particle size of niosomes. Similar pattern in particle size was observed in case of drug loaded niosomal formulation (as discussed by Sambhakar et al. [43]). Mean vesicles' size of drug loaded niosomes was found to be greater than the unloaded niosomes at each ratio of drug : cholesterol : surfactant with different grade of Span (20, 40, 60 and 80).

### 5.4. Entrapment Efficiency

**Figure 4:**
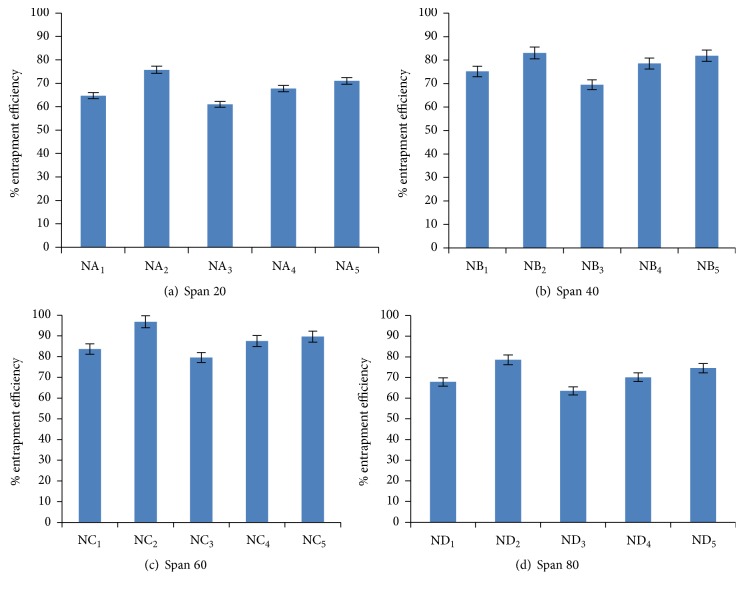
Entrapment efficiency of niosomes containing different series of Span at various ratios.

Entrapment efficiency is the percentage fraction of the entire drug entrapped in the niosomes. Entrapment efficiency of niosomal formulation was determined by centrifugation method and result was displayed in Figure 4. Entrapment efficiency of drug loaded niosomal formulation was found to be increased on increasing the cholesterol ratio from 0.5 to 1 whereas entrapment efficiency decreases on further increase in cholesterol ratio from 1 to 1.5. This might be due to two factors. First, with increase cholesterol ratio, hydrophobicity and stability of bilayers vesicles increase and permeability decrease which may lead to efficiently trapping the hydrophobic drug into bilayers as the vesicles formed. Secondly, higher amount of cholesterol may compete with the drug for packing space within the bilayer hence excluding the drug as the amphiphiles assembled into drugs (as discussed by Balakrishnana et al. [35]).

**Figure 5:**
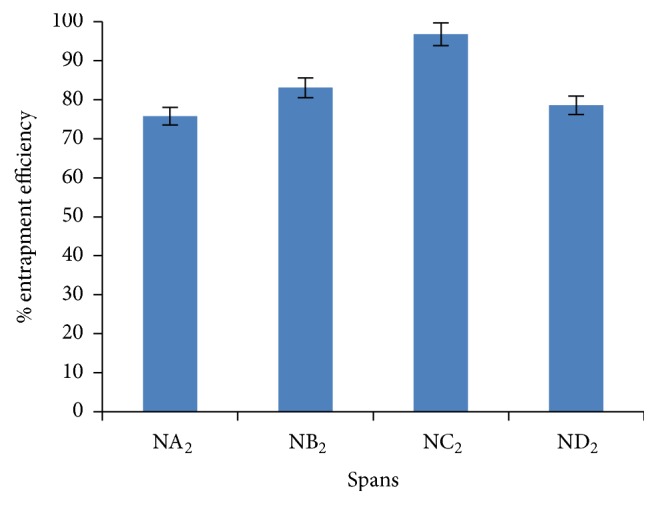
Comparative encapsulation efficiency of various series of niosomal formulation at ratio 1 : 1 : 1 (drug : cholesterol : surfactant).

Further, comparing the various niosomal formulations containing different grade of Span (Span 20, Span 40, Span 60, and Span 80) at 1 : 1 ratio of surfactant to cholesterol (Figure 5), Span 60 containing niosomal formulation (NC_2_) displayed highest entrapment efficiency as compared to other formulations. This might be due to the fact that Span 60 has longest alkyl chain length compared to other Span series. Entrapment efficiency of all niosomal formulations with different grade of Span was found in the following order:

(4) $$\begin{array}{c} \text{Span }60>\text{Span }40>\text{Span }80>\text{Span }20. \end{array}$$

Similar pattern in entrapment efficiency of minoxidil containing niosomes of different Span grade was reported (as discussed by Balakrishnana et al. [35]).

From the above data, niosomal formulation having highest entrapment efficiency in all Span series was selected for further studies.

### 5.5. *In Vitro *Release Studies

**Figure 6:**
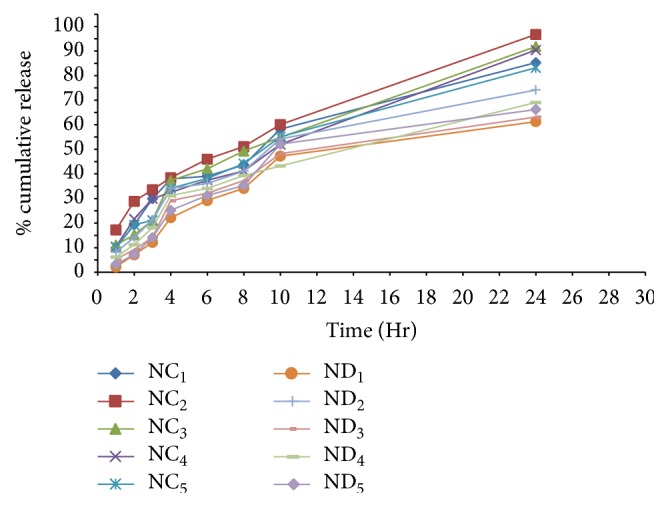
Comparative release profile of selected niosomal formulations.

Particle size ranges less than 5 *μ*m are required for passive delivery of drug through alveolar region (as discussed by Bi and Zhang [44]). On the basis of particle size and entrapment efficiency, suitable niosomal formulation was selected for* In vitro* drug releases' studies.* In vitro *studies of selected formulations were carried out in PBS pH 7.4 by dialysis method on magnetic stirrer and results were shown in Figure 6. It was clearly observed from the data as shown in Figure 6 that* in vitro* drug release of niosome containing Span of different series (60 and 80) was sharply increased up to 24 hr. Maximum drug release, that is, 96.78%, was reported in case of niosome containing Span 60 as compared to other series of Span 80 after 24 hr as shown in Figure 6. This might be due to longest alkyl chain length of Span 60 and thus possesses highest release profile (as discussed by Sambhakar et al. [43]). In contrast, Span 80 has monounsaturated alkyl chain and thus has lowest release profile compared to Span 60.

### 5.6. Zeta Potential and Polydispersity Index

On the basis of result of above data, NA_2_, NB_2_, NC_2_, and ND_2_ niosomal formulation were selected for further studies and zeta potential was further confirmed by using Zetasizer. It was clearly observed from the data as shown in Table 3 that highest zeta potential was observed with Span 20 whereas lowest zeta potential was in case of Span 60. This might be due to increases in hydrophilicity of surfactant; zeta potential also increases (as discussed by Balakrishnana et al. [35] and Sambhakar et al. [43]). Polydispersity index of selected formulation of Span 20, Span 40, Span 60, and Span 80 was found to be 0.319, 0.263, 0.233, and 0.279, respectively, indicating the homogeneity of the formulations.

### 5.7. Cell Cytotoxicity Study

**Figure 7:**
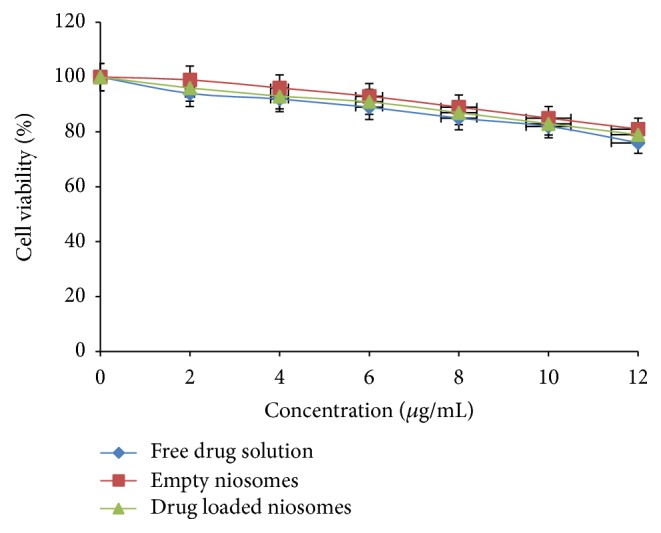
Cell viability study of free drug solution; empty niosomes; drug loaded niosomes on A549 cell lines (*n* = 3).

The percent viability of the same concentration of drug loaded niosomes was determined after exposure to A549 cells. The same concentration of free drug solution was also used for comparison. Empty niosomal formulation and drug loaded niosomes exhibited lower cytotoxicity as compared to free drug solution as shown in Figure 7. It might be due to the different cellular uptake of niosomal CLR and the free form of the drug. According to these preliminary toxicity results, it can be concluded that drug niosomal formulations exhibited less cytotoxicity towards alveolar epithelial cells compared to free drug solution. The components used in the formulations were generally regarded as safe.

Further, according to USFDA inactive ingredients approval list, the maximum potency of Span 60 which is nontoxic in nature, in suspension form, is 62.5 mg/5 mL and cholesterol of sterile formulation lies in range from 0.03% to 0.33%w/w and LD50 value for Span 60 is 15.9 g/kg (MSDS safety report). Developed niosomal formulation contained Span 60 equivalent to 5 mg/mL and thus was found to be safer range for lung tissues. Similarly, cholesterol was administered in niosomal formulation which was equivalent to 5 mg/mL and found to be in safer range.

### 5.8. Stability Study

On the basis of result of particle size, percentage entrapment efficiency,* in vitro *release profile, zeta potential, and polydispersity index, (NC_2_) was selected for further stability studies and* in vivo *study. Stability study of NC_2_ formulation was carried out by keeping the formulation at storage conditions (refrigerated temperature and room temperature) and their stability was determined with respect to particle size and residual drug content at the 7th, 14th, 21st, 28th, and 30th days' interval.

The particle size of the niosomes was slightly increased at room temperature while there was no significant change observed at refrigerated temperature as shown in Table 4. Further encapsulation efficiency slightly decreased at room temperature, whereas, in case of refrigerated temperature, no significant change was observed in entrapment efficiency as shown in Table 5. NC_2_ formulation was found to be stable at the end of the study on storage condition.

### 5.9. *In Vivo* Studies

On the basis of result of* in vitro* characteristics,* in vitro* release studies, and stability studies, NC_2_ formulation was selected for* in vivo* studies. The concentration of drug in plasma at different time interval after pulmonary administration to different group of SD rats was determined. Different pharmacokinetic parameters such as *t*_max_, area under curve (AUC), area under mean curve (AUMC), and mean residence time (MRT) were calculated from data and results were represented in Table 6. *C*_max_ achieved by plain CLR was 2.488 *μ*g/mL, while in case of CLR-entrapped niosomes *C*_max_ was found to be 4.321 *μ*g/mL. There was significant difference in concentration of both formulations at 4 h. After 24 hr, 2.011 *μ*g/mL concentration of drug remains present in blood with NC_2_ formulation, whereas only 0.823 *μ*g/mL concentration was obtained in case of plain drug. Various pharmacokinetic parameters, namely, AUC, AUMC, and MRT of niosomal formulation, were found to be 1.5-fold, 4-fold, and 3-fold plain drug, respectively. This indicates that niosomal formulation provides sustained and prolonged delivery of drug.

## 6. Conclusion

In the present study, the findings revealed that the process variables critically affect the formulation of niosomes with regard to drug loading and need to be carefully controlled. In conclusion, our study suggests that these niosomal formulations provide sustained and prolonged delivery of drug with enhanced bioavailability. The niosomal formulation through pulmonary route could be a useful dosage form to reduce the undesirable side effects associated with oral route.

## Figures and Tables

**Table 1:** Composition of empty niosomal formulations at various ratios of cholesterol : surfactant.

Sr. number	Formulation codes	Drug : cholesterol : surfactant	Dicetyl phosphate (DCP) (mg)	Surfactant grade
1	NA_1_	1 : 0.5 : 1	5.0	SPAN-20
2	NA_2_	1 : 1 : 1	5.0	SPAN-20
3	NA_3_	1 : 1.5 : 1	5.0	SPAN-20
4	NA_4_	1 : 1 : 0.5	5.0	SPAN-20
5	NA_5_	1 : 1 : 1.5	5.0	SPAN-20
6	NB_1_	1 : 0.5 : 1	5.0	SPAN-40
7	NB_2_	1 : 1 : 1	5.0	SPAN-40
8	NB_3_	1 : 1.5 : 1	5.0	SPAN-40
9	NB_4_	1 : 1 : 0.5	5.0	SPAN-40
10	NB_5_	1 : 1 : 1.5	5.0	SPAN-40
11	NC_1_	1 : 0.5 : 1	5.0	SPAN-60
12	NC_2_	1 : 1 : 1	5.0	SPAN-60
13	NC_3_	1 : 1.5 : 1	5.0	SPAN-60
14	NC_4_	1 : 1 : 0.5	5.0	SPAN-60
15	NC_5_	1 : 1 : 1.5	5.0	SPAN-60
16	ND_1_	1 : 0.5 : 1	5.0	SPAN-80
17	ND_2_	1 : 1 : 1	5.0	SPAN-80
18	ND_3_	1 : 1.5 : 1	5.0	SPAN-80
19	ND_4_	1 : 1 : 0.5	5.0	SPAN-80
20	ND_5_	1 : 1 : 1.5	5.0	SPAN-80

**Table 2:** Solubility of CLR in different solvents (*n* = 3).

Sr. number	Solvents	Solubility (mg/mL)
1	PBS 7.4	0.11 ± 0.005
2	Acetone	74 ± 1.2
3	Methanol	67 ± 0.2
4	Ethanol	63 ± 0.2
5	Dichloromethane	221 ± 1.4
6	Chloroform	18 ± 0.6

**Table 3:** Zeta potential and polydispersity index.

Sr. number	Formulation	Zeta potential	Polydispersity index	Particle size (*μ*m)
1	NA_2_	−31.12	0.319 ± 0.052	7.972 ± 0.019
2	NB_2_	−29.67	0.263 ± 0.039	7.099 ± 0.041
3	NC_2_	−24.93	0.233 ± 0.091	4.674 ± 0.028
4	ND_2_	−26.62	0.279 ± 0.044	4.449 ± 0.070

**Table 4:** Effect of storage on particle size at refrigerated and room temperature.

Formulation code	Days interval	Particle size (*μ*m)		
		Initial	Refrigerated temperature	Room temperature
NC_2_	7th day	4.674 ± 0.028	4.681 ± 0.021	4.711 ± 0.037
	14th day		4.703 ± 0.019	4.809 ± 0.062
	21st day		4.741 ± 0.040	4.894 ± 0.044
	28th day		4.793 ± 0.029	5.014 ± 0.057
	30th day		4.871 ± 0.033	5.331 ± 0.041

**Table 5:** Effect of storage on percentage residual content at refrigerated and room temperature.

Formulation code	Days interval	Initial percentage	Refrigerated temperature	Room temperature
NC_2_	7th day	96.78 ± 0.054	95.82 ± 0.023	93.21 ± 0.027
	14th day		94.43 ± 0.031	91.32 ± 0.034
	21st day		93.79 ± 0.017	88.29 ± 0.048
	28th day		90.69 ± 0.044	84.58 ± 0.031
	30th day		89.92 ± 0.051	82.39 ± 0.019

**Table 6:** Pharmacokinetic parameter of drug determined after administered of plain drug and niosomal formulation.

Sr. number	Parameters	Free drug	NC_2_
1	*C*_max_	2.488 *μ*g/mL	4.321 *μ*g/mL
2	*t*_max_	6 hr	4 hr
3	AUC	24.50 *μ*g/mL·hr	35.50 *μ*g/mL·hr
4	AUMC	160 *μ*g/mL·hr^2^	670 *μ*g/mL·hr^2^
5	MRT	6.66 hr	18.87 hr
